# Genomics and Virulence of *Klebsiella pneumoniae* Kpnu95 ST1412 Harboring a Novel Incf Plasmid Encoding *Bla*ctx-M-15 and *Qnrs1* Causing Community Urinary Tract Infection

**DOI:** 10.3390/microorganisms9051022

**Published:** 2021-05-10

**Authors:** Ayala Gancz, Kira Kondratyeva, Dorit Cohen-Eli, Shiri Navon-Venezia

**Affiliations:** 1 Molecular Biology Department, Faculty of Life Sciences, Ariel University, Ariel 40700, Israel; ayalagancz@gmail.com (A.G.); lutra007@gmail.com (K.K.); dorit553@gmail.com (D.C.-E.); 2 The Miriam and Sheldon Adelson School of Medicine, Ariel University, Ariel 40700, Israel

**Keywords:** ESBL, *Klebsiella pneumoniae*, sequence type 1412 (ST1412), genomics, community urinary tract infection, *bla*_CTX-M-15_, *qnrS1*, persistome, *Caenorhabditis elegans*

## Abstract

The emergence of extended-spectrum β-lactamase (ESBL)-producing multidrug resistant *Klebsiella pneumoniae* causing community urinary tract infections (CA-UTI) in healthy women undermines effective treatment and poses a public health concern. We performed a comprehensive genomic analysis (Illumina and MinION) and virulence studies using *Caenorhabditis elegans* nematodes to evaluate KpnU95, a *bla*_CTX-M-15_-producing CA-UTI *K. pneumoniae* strain. Whole genome sequencing identified KpnU95 as sequence type 1412 and revealed the chromosomal and plasmid-encoding resistome, virulome and persistence features. KpnU95 possess a wide virulome and caused complete *C. elegans* killing. The strain harbored a single novel 180.3Kb IncFIB(K) plasmid (pKpnU95), which encodes ten antibiotic resistance genes, including *bla*_CTX-M-15_ and *qnrS1* alongside a wide persistome encoding heavy metal and UV resistance. Plasmid curing and reconstitution were used for loss and gain studies to evaluate its role on bacterial resistance, fitness and virulence. Plasmid curing abolished the ESBL phenotype, decreased ciprofloxacin MIC and improved bacterial fitness in artificial urine accompanied with enhanced copper tolerance, without affecting bacterial virulence. Meta-analysis supported the uniqueness of pKpnU95 and revealed plasmid-ST1412 lineage adaptation. Overall, our findings provide translational data on a CA-UTI *K. pneumoniae* ST1412 strain and demonstrates that ESBL-encoding plasmids play key roles in multidrug resistance and in bacterial fitness and persistence.

## 1. Introduction

Extended-spectrum β-lactamase (ESBL)-producing *Klebsiella pneumoniae* is a worldwide opportunistic bacteria belonging to the ESKAPE group [1]. This pathogen poses a huge burden in human medicine due to its multidrug resistance (MDR) phenotype leading to limited treatment options [2]. Classical hospital-associated *K. pneumoniae* infections are pneumonia, bacteremia, urinary tract and wound infections [3,4], mostly affecting immunocompromised patients [5].

*K. pneumoniae* is considered as the second most common uropathogen after *Escherichia coli* [6]. The emergence of this species in the community setting is disturbing due to its high antibiotic-resistant nature compared to *E. coli,* mainly due to the production of ESBLs [5,7]. Multiple studies describe the molecular epidemiology of ESBL-producing *K. pneumoniae* clones in acute and long-term care facilities [8]. However, there is limited knowledge on the genomics and the pathogenesis of community strains [9].

All *K. pneumoniae* possess a subset of core chromosomal encoded pathogenicity factors including adhesion, capsule production and iron uptake [3,4]. This core virulence repertoire can be extended with additional virulence features such as extra siderophores, mucoid production regulators, invasion mechanisms, nutrient acquisition and protection from the human immune system leading to an enhanced virulence phenotype [4,10,11].

The genetic repertoire of ESBL-producing *K. pneumoniae* can be drastically extended by the presence of MDR virulence-encoding megaplasmids [12,13]. We hypothesize that *K. pneumoniae* strains that successfully cause urinary tract infections (UTI) in healthy young women without apparent risk factors should possess enhanced virulence and environmental persistence. Deep genetic characterization of ESBL-producing *K. pneumoniae* strains, which cause community infections in the healthy population, is highly important in order to track the spread of antibiotic resistance and virulence genes in the community and to optimize empiric antibiotic therapy.

In this study, using whole genome sequencing (WGS), antibiotic susceptibility testing, fitness studies and virulence assessment using the host–pathogen *Caenorhabditis elegans* model, we characterized KpnU95, a CA-UTI ESBL-producing *K. pneumoniae* strain. We describe the virulome and the resistome of the KpnU95 strain and the complete sequence of a novel megaplasmid that encodes a wide resistome and multiple metal and UV resistance genes, which may contribute to the bacterial environmental persistence. Furthermore, we describe a meta-analysis with a collection of available *K. pneumoniae* ST1412 genomes and demonstrate the co-existence of this clonal lineage with the MDR megaplasmid.

## 2. Materials and Methods

### 2.1. K. pneumoniae UTI Strain, Growth Conditions and Antibiotic Susceptibility Testing

*K. pneumoniae* strain U95 (KpnU95) was isolated from a positive urine culture collected from a healthy young woman in 2016 during an outpatient UTI study we performed in Israel [14]. All ESBL-producing isolates in this study were routinely grown in Luria broth (LB) media or LB agar plates containing ampicillin (100 μg/mL) to maintain the natural antibiotic-encoding plasmids. Antibiotic susceptibility testing was performed using Sensititre™ Gram Negative GN4F AST Plates (Thermo Scientific™) following the manufacturer’s instructions. ESBL production was confirmed using a clavulanic acid combination disc assay (Oxoid, BD). Ciprofloxacin MICs were determined with Etest (bioMérieux, Marcy-l’Étoile, France). All MICs were interpreted according to the EUCAST susceptibility testing breakpoints [15].

### 2.2. Purification of the ESBL-Encoding Plasmid and Transformation

The ESBL-encoding plasmid - pKpnU95 was purified from the clinical KpnU95 strain using the Plasmid Midi Kit (QIAGEN, Hilden, Germany) following the manufacturer’s instructions. The purified plasmid was then electroporated into competent *E. coli* DH10B recipient strain (Invitrogen, Carlsbad, CA, USA). Transformants were selected on LB agar plates containing 100 μg/mL ampicillin (Gold-bio) and additionally screened for the presence of the *bla*_CTX-M group-1_ gene [16]. Plasmid purification was repeated from the transformant to confirm plasmid purity. Antibiotic susceptibility testing of the transformant strain carrying pKpnU95 was performed using the Vitek-2 (bioMérieux, Marcy-l’Étoile, France) and Sensititre to determine the acquired resistance profile after plasmid acquisition.

### 2.3. Curing of pKpnU95 and Plasmid Reconstitution

KpnU95 clinical strain was cured from pKpnU95, its ESBL-encoding plasmid, in rich BHI medium with once-a-day sequential passages in fresh BHI at 42ºC as previously described [17]. Cured colonies lacking pKpnU95 were isolated after 21 days using replica plating onto LB plates with and without ceftriaxone (4 μg/mL). The isogenic nature of the clinical and the cured strain after passages was verified using a modified Enterobacterial repetitive intergenic consensus sequences (ERIC)-PCR method [18]. Loss of pKpnU95 in the cured strain was confirmed by PCR reactions verifying the absence of *bla*_CTX-M_ group-1 and *qnrS1* genes and further using MinION sequencing. Antibiotic susceptibility testing of the cured strain was performed using the VITEK^®^2 system together with combination disc assay and MIC determination for ciprofloxacin (Etest).

To construct a reconstituted clinical strain, the cured strain was transformed with purified pKpnU95 DNA using electroporation. The transformant colonies were selected on LB plates supplemented with ceftriaxone (4 μg/mL) after overnight incubation at 37 °C. In parallel, conjugation experiments of the ESBL-encoding plasmid pKpnU95 were performed according to a previously described method [17] using the filter mating method.

### 2.4. Fitness Studies

Fitness studies were performed on the three *K. pneumoniae* strains that we constructed: KpnU95 clinical strain, KpnU95 cured strain and the cured strain transformed with pKpnU95 plasmid. Bacterial growth experiments were performed in LB medium, basal minimal medium 2 (BM2) [19] and in artificial urine [20]. Furthermore, growth experiments in the presence of CuSO_4_ were conducted as described previously [21], with minor modifications. The strains were grown in Mueller Hinton (MH) or LB containing ampicillin (100 μg/mL). Absorbance measurements monitored bacterial growth at 600 nm in 96-multiwell plates placed in an automated plate reader (Infinite M200 PRO, Tecan, Männedorf, Switzerland) at 37 °C for 14 h. Growth curves were generated from three individual experiments and the A_600nm_ measurements were averaged from six duplicate wells. Raw data analysis and growth parameters (growth rate and yield) were calculated using an Infianalysis algorithm application that was developed in our lab. Statistical analysis was performed using Student’s *t*-test using Prism 8 (GraphPad Software, San Diego, CA, USA) with *p* ≤ 0.05 considered as significant.

### 2.5. Virulence Assessment in the Caenorhabditis Elegans Host–Pathogen Model

The virulence of KpnU95 was evaluated by performing killing assays of *C. elegans* nematodes fed on the bacteria using a liquid-based assay, as described previously [22]. *C. elegans* AU98 strain, purchased from the Caenorhabditis Genomics Center (CGC) (Minneapolis, MN, USA), was used for the killing assays and *E. coli* strain OP50 (CGC, Minneapolis, MN, USA) was included as the negative non-virulent control bacterial strain. The nematode killing assays were conducted with synchronized L4-stage AU98 nematodes in 384-multiwell plates (Greiner Bio-One, Kremsmünster, Austria). The virulence of KpnU95 was assessed in two different minimal media, BM2 and artificial urine. Nematode killing assays in the presence of each of the tested *K. pneumoniae* strain were performed using a high content/high throughput microscope (Hermes^®^ WiScan instrument, Rehovot, Israel) equipped with Athena software (IDEA Bio-Medical Ltd) and FIJI software [23], to quantify live or dead nematodes. Statistical analysis in the nematode killing assays was performed using Log-rank test using Prism 8 (GraphPad Software, San Diego, CA, USA) and *p* ≤ 0.05 was considered as significant.

### 2.6. WGS of KpnU95

KpnU95 DNA for WGS was purified using DNeasy^®^ Blood & Tissue Kit (QIAGEN, Hilden, Germany), according to the manufacturer’s instructions with minor modifications, in which the bacterial pellet was additionally washed with nuclease-free water prior to purification and DNA was eluted with Tris-HCl-EDTA-free buffer (pH 7.5). WGS was performed using Illumina MiSeq (Illumina, San Diego, CA, USA) at the Technion Genome Center (Technion Institute of Technology, Haifa, Israel) using a 2 × 250 bp paired-end libraries. Following WGS, adapter sequences and low-quality reads were eliminated using Trimmomatic-0.36 [24] and assembly was performed using SPAdes-3.11.1 with an algorithm for assembling long Illumina paired reads [25].

### 2.7. Complete Sequencing of Plasmid pKpnu95

To complete and validate pKpnU95 assembly from the WGS data, the purified plasmid (see Section 2.2) was additionally sequenced using long-read Nanopore technology (Oxford Nanopore Technologies, ONT, Oxford, United Kingdom) following the standard protocol SQK-RAD004 v. RSE_9046_v1_revB_17Nov2017. Plasmid library was prepared from 200 ng purified plasmid DNA using the SQK-RBK004 ONT Rapid barcoding sequencing kit and loaded onto the MinION flow cell FLO-MIN106 (R9.4 SpotON) for a 6-hr run. Base-calling was performed by albacore v2.3.3 and adapter sequences were removed by Porechop v0.2.3. The hybrid read set of Illumina and Nanopore reads were assembled into a single circular plasmid using Unicycler v0.4.0 [26].

### 2.8. Development of a Targeted PCR Screening for the Presence of pKpnu95

Based on the complete sequence of pKpnU95, we developed a specific 7-gene-based PCR for screening the presence of pKpnU95. The PCR was based on seven genes that are distributed evenly on the plasmid. The genes (*bla*_CTX-M-1_, *umuD*, IncFIB (K), *qnrS1*, *hisP*, *chrA* and *pcoB*) and the respective primers used to amplify them are described in Table S3. We used this pKpnU95-targeted PCR to screen the transformants in order to confirm the acquisition of pKpnU95.

### 2.9. Bioinformatics Analysis

KpnU95 WGS scaffolds were annotated by NCBI Prokaryotic Genome Annotation Pipeline [27] and plasmid pKpnU95 was annotated using RAST [28]. Predicted proteins were verified and updated using Swiss-Prot/UniProtKB database. CGE pipeline tools were used to identify ARGs (ResFinder-2.1), virulence genes (VirulenceFinder-1.2), to perform in silico multi-locus sequencing typing (MLST-2.0) and plasmid typing (PlasmidFinder-1.2, pMLST-1.4). Plasmid partition gene typing was performed in silico [29]. *parC* binding sites (16 bp repeats) were predicted in the *parB* downstream area using BLASTn. OriT and conjugation genes were searched using OriTDB database and online OriTfinder [30]. IS elements were found by ISfinder and class I integron was assigned by INTEGRALL. The position of IS26 transferable element was determined by BLASTn-search. *K. pneumoniae* locus typing was performed by Kaptive [31].

#### 2.9.1. GenBank Submission

KpnU95 WGS data were submitted to the GenBank (BioProject PRJNA494961) and the complete sequence of pKpnU95 was deposited at the NCBI Nucleotide database (GenBank accession number MK552109).

#### 2.9.2. Analysis of pKpnU95-Closely Related Plasmids

pKpnU95 related plasmids were mined from the Nucleotide (nt/nr) collection using BLASTn-based search for homologous sequences to the complete pKpnU95 sequence. In addition we searched for pKpnU95 IncFIK-carrying backbone region (19808 bp, location—join(177146..180286,1..16667)).

Further, to identify pKpnU95-related plasmids from *K. pneumoniae* WGS data, we restricted our search to NCBI BioProjects, which contained SRA data described as ‘*K. pneumoniae* ST1412’. The retrieved Illumina short-reads sequencing data of SRA *K. pneumoniae* isolates from the selected BioProjects were mapped to pKpnU95 using bwa v0.7.17-r1188 and were analyzed using samtools-1.7 and bcftools-1.8.

The filtration criteria for homologous sequences were 100% pKpnU95 backbone coverage for both Nucleotide complete sequenced plasmids and SRA isolates. For the visualization of plasmids, the mapped reads were converted into pseudo-sequences using samtools mpileup output results. Plasmid map was generated using BRIG-0.95 [32].

#### 2.9.3. Phylogenetic Analysis of pKpnU95-Closely Related Plasmids

A phylogenetic tree was generated for all pKpnU95-related plasmids. Full-length alignments of SRA isolates and the complete sequenced plasmids, to pKpnU95 were generated by snippy v4.3.6 (https://github.com/tseemann/snippy, accessed on 4 August 2019), using a minimum coverage of five reads and base-call stringency of 80% adjusting for low coverage depth of several Illumina sequencing data. Plasmid recombination regions were masked by gubbins v2.3.4 [33] and maskrc-svg (https://github.com/kwongj/maskrc-svg, accessed on 4 August 2019). A maximum-likelihood phylogenetic tree with 1000 ultrafast bootstrap support was constructed using IQ-TREE v1.6.12 [34] and visualized with iTOL v4 [35].

## 3. Results

### 3.1. Kpnu95 Isolate and Generation of a Cured and a Reconstituted Cured Strain

*K. pneumoniae* KpnU95 was isolated from a positive urine culture from a healthy young woman with a community UTI [14]. The isolate possessed an MDR ESBL phenotype including resistance to all cephalosporins and trimethoprim/sulfamethoxazole and elevated MICs to quinolones (Table 1). Based on the WGS data (presented later on, in Section 3.4), KpnU95 carried a single ESBL-encoding plasmid, designated pKpnU95.

In order to evaluate the role of pKpnU95 on KpnU95 resistance profile, fitness and virulence, we used an elimination and a reconstitution approach. For this, we cured the clinical parent strain KpnU95 from pKpnU95 to generate KpnΔpKpnU95 and then reconstituted the cured strain by transforming pKpnU95 to generate KpnΔpKpnU95/pKpnU95. We confirmed the elimination and the reconstitution of pKpnU95 using a 7-gene targeted PCR that we developed based on the plasmid complete sequence (Section 3.4.2 and Figure S1). The cured and the reconstituted strains possessed an identical ERIC pattern, which confirmed that they were isogenic to the parent KpnU95 strain (Figure S2). In addition, we generated an *E. coli* DH10B transformant that had acquired pKpnU95 plasmid.

### 3.2. Antibiotic Susceptibility

**Figure 1:**
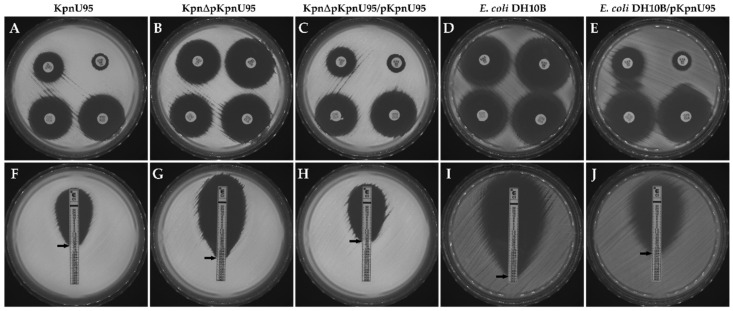
The effect of pKpnU95 on the ESBL phenotype and ciprofloxacin (CIP) MIC testing. ESBL confirmation assay using ceftazidime (CAZ); upper-left disc, ceftazidime (CAZ); lower-left CAZ + clavulanic acid (CLA); upper-right, cefotaxime (CTX); and lower-right CTX + CLA (**A**–**E**); and Etest MIC testing of ciprofloxacin (CIP) (**F**–**J**). The clinical KpnU95 (**A**,**F**), the cured strain lacking the plasmid, KpnΔpKpnU95 (**B**,**G**), the cured strain transformed with the plasmid, KpnΔpKpnU95/pKpnU95 (**C**,**H**), *E. coli* DH10B (**D**,**I**) and *E. coli* DH10B/pKpnU95 (**E**,**J**). Images are representative of three biological replicates.

Antibiotic susceptibility profiles showed that curing of pKpnU95 plasmid from the clinical KpnU95 strain resulted in loss of the MDR phenotype and turned KpnΔpKpnU95 susceptible to all cephalosporins and monobactams and to trimethoprim/sulfamethoxazole (Table 1). The reconstituted strain KpnΔpKpnU95/pKpnU95 showed a similar resistance profile as the clinical parent KpnU95. We observed a reversal of the ESBL phenotype and a decrease of 11.8-fold in ciprofloxacin MIC upon curing. The *E. coli* DH10B transformant carrying pKpnU95 displayed an ESBL phenotype, similar to the clinical KpnU95 strain, together with a 32-fold increase in MIC to ciprofloxacin (Table 1 and Figure 1). All the KpnU95 strains showed the same level of non-susceptibility to nitrofurantoin compared to *E. coli* DH10B and *E. coli* DH10B/pKpnU95 that were nitrofurantoin sensitive, suggesting that this non-susceptibility is chromosomally encoded (Table 1).

### 3.3. Fitness and In Vivo Virulence of K. pneumoniae KpnU95 Strains

**Figure 2:**
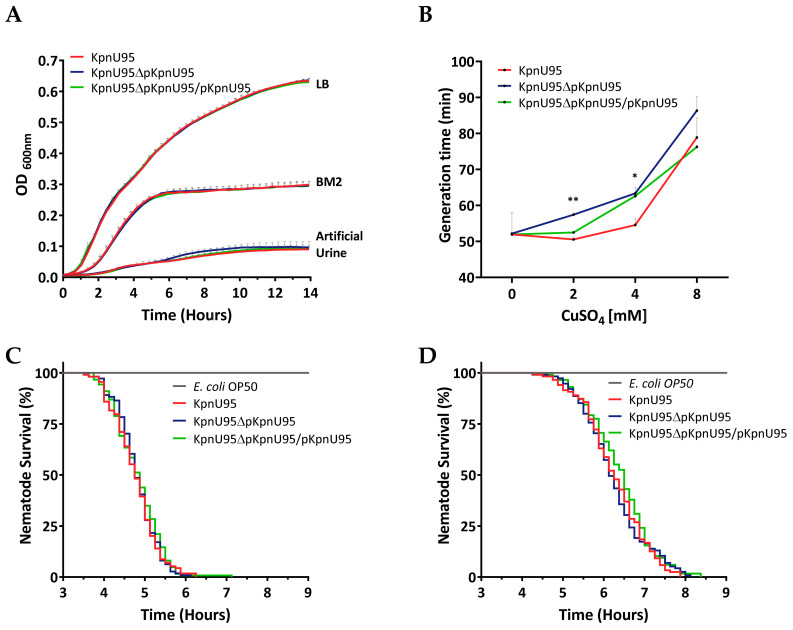
Growth kinetics, copper tolerance and virulence assessment of KpnU95 strains. Growth curves of KpnU95 strains in rich media LB, minimal BM2 medium and on artificial urine were performed in 96-multiwell plates by monitoring OD600 nm during 14 h. Each growth curve represents the average of three or five independent experiments (**A**); Copper tolerance in MH in different concentrations of CuSO_4_. The doubling time of each growth curve represents the average of three independent growth experiments. * *p* value ≤ 0.05 and ** *p* value ≤ 0.01 (**B**); The survival curves of C. elegans nematodes fed on KpnU95 strains or *E. coli* OP50 as a control strain in BM2 (**C**) or on artificial urine media (**D**). The nematode survival curves were plotted according to the average survival counts of two replicate experiments performed in four replicate wells. Statistical analysis was performed using the Log-rank (Mantel–Cox) test (**C**,**D**).

To evaluate the contribution of the ESBL-encoding plasmid pKpnU95 on KpnU95 fitness and virulence, we evaluated and compared the fitness and virulence of KpnU95, its cured derivative and the cured reconstituted strain. Fitness experiments showed that the growth kinetics and the average generation time between the three strains was similar in the three different media that they were grown on but fitness cost was observed only when the strains grew on artificial urine (Figure 2A and Table S1). Although the growth of all the three strains was poor in artificial medium, the cured strain grew slower than the clinical strain with a longer generation time (103.85 ± 2.68 min compared to 95.37 ± 2.09 min, *p* value = 0.0361, Table S1), proving that pKpnU95, the ESBL-encoding plasmid, contributes to growth under these conditions.

Growth of KpnU95 harboring pKpnU95 in the presence of copper ions was faster than the cured strain lacking the plasmid (doubling time of 50.5 vs. 57.4 min at 2 mM CuSO_4_, *p* value = 0.008 and 54.6 vs. 63.3 min at 4 mM CuSO_4_, *p* value = 0.04, Table S2). These growth differences demonstrate the contribution of pKpnU95 to copper tolerance.

Virulence assessment of KpnU95 strains in the *C. elegans* host–pathogen model demonstrated complete nematode killing by the three strains compared to *E. coli* OP50, the a-virulent control strain (*p* value < 0.0001). Virulence of KpnU95 strains was observed on both minimal media (Figure 2B) and artificial urine, in spite of the poor growth (Figure 2C). The lethal time 50 (LT50) values of the nematodes by KpnU95, KpnU95 cured strain and the reconstituted cured strain were very similar, proving the lack of involvement of pKpnU95 plasmid in the nematode killing. The kinetics of virulence was influenced by the media the nematodes were grown on, with faster killing in BM2 (LT50 of 4.75 h, 4.75 h and 4.88 h, respectively) compared to the killing rate measured in artificial urine medium (LT50 of 6.25, 6.12 and 6.5 h, respectively), *p* value < 0.0001, presumably due to superior bacterial growth.

### 3.4. Genomic Characterization of KpnU95

In silico WGS analysis and assembly revealed that KpnU95 belonged to ST1412 lineage with a K109 capsular type harboring the ESBL-encoding plasmid, pKpnU95.

#### 3.4.1. KpnU95 Chromosome

KpnU95 chromosome size was 5,055,295 bp, encoding 5087 ORFs with 57.76% G+C content. The accessory genes of KpnU95 were classified into three main categories: (i) virulence factors that are involved in bacterial pathogenesis and survival within the host; (ii) persistence genes important for environmental persistence, resistance to toxic substances and stress response; and (iii) antibiotic resistance genes (Table 2). KpnU95 virulome consisted of multiple host–pathogen interacting systems, including adhesion mechanisms and biofilm formation, growth regulators via six toxin–antitoxin systems and metabolic pathways, including iron acquisition systems and urea/allantoin digestion, together with two secretion systems. The majority of the persistence genes encoded metal transport systems and the UV resistance DNA repair locus, *umuCD* (Table 2).

KpnU95 chromosome encodes a wide resistome conferring MDR phenotype to multiple antibiotic families and detergents (Table 2). The strain encoded multiple efflux pumps, including *oxqAB,* which may confer low or intermediate resistance to quinolones, nitrofurantoin and several detergents. In addition, KpnU95 also encoded the oxygen-insensitive NADPH nitroreductase showing 75.62% identity to *nfsA* (NC_000913.3) that may catalyze the decline of nitroaromatic compounds using NADPH [37]. The chromosomal co-existence of this gene may explain the resistance to nitrofurantoin (Table 1) [38].

#### 3.4.2. Complete Sequence of pKpnU95

pKpnU95 was an IncF megaplasmid (180,286 bp), encoding 243 ORFs, with 50.21% G+C content. The plasmid encodes an IncFIB(K) replicon that showed 100% identity to *Klebsiella oxytoca* pCAV1099-114 replicon locus (accession number CP011596) and belonged to F4 partition system type. Downstream to *parB* gene, *parS* binding area was predicted by the presence of the repeating 16 bp sites (TGGGACCACGGTCCCA). pKpnU95 encodes a wide resistome consisting of 10 ARGs, including the *bla*_CTX-M-15_ ESBL gene. The presence of *qnrS1* on the plasmid explains the differences in ciprofloxacin MICs between plasmid-carrying and plasmid-free strains (Table 1).

**Figure 3:**
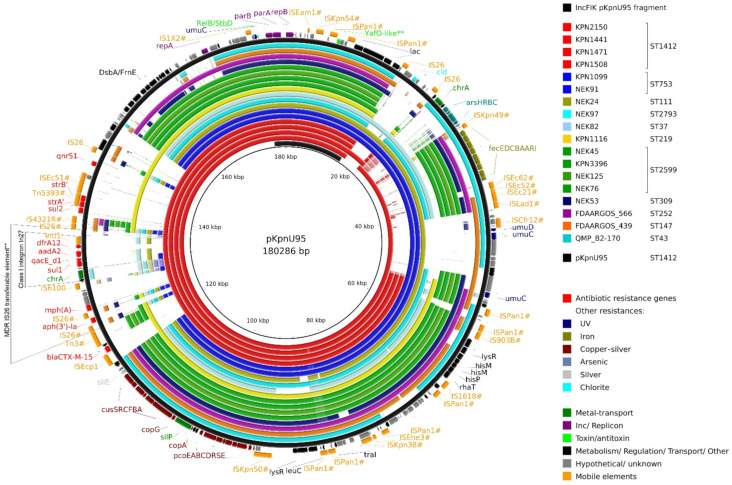
Comparison of pKpnU95 and homologous *K. pneumoniae* plasmids. A BRIG diagram shows pKpnU95 (black) and alignments of the 18 related plasmids, designated in different colors according to their STs. pKpnU95 backbone region is presented on the inner circle (black segment). The annotation of pKpnU95 is shown on the two outer rings representing the genes and the mobile elements as strand-oriented arrows. IS elements and transposons labeled with ‘#’ showed >90% identity and >30% coverage of the sequences from the ISfinder database. Truncated transposons may not encode all expected ORFs, but only part of them. ** Computationally predicted only.

The analysis of ARG cassettes revealed the presence of a class I integron In27 that encodes *intI1-dfrA12-aadA2-qacEΔ1-sul1-chrA-orf*, incorporated into an IS26 transferable element. This element (MK552109, 122905-137024) was found in other *K. pneumoniae* and Enterobacterales plasmids (>99% coverage and >99% sequence identity). This suggests the simultaneous transfer of an IS26 transferable element consisting of IS26#–*mph(A)*–orf (MFS transporter)–orf (TetR/AcrR family transcriptional regulator)–orf–IS6100–orf–class I integron. This unit started with an altered IS26 (Figure 3, labeled as IS26#) that shared 99% identity with IS26 and also differed from the IS26# located at the opposite side of the element. Taken together, the sequence difference between the two IS26 that flank the region and the fact that this element was not found in the database with intact IS26 on both sides suggests that this MDR IS26 transferable element originated from a translocatable unit with a single IS26.

In pKpnU95, an IS26 transferable element was surrounded by truncated Tn3-family transposons (Figure 3; Tn5393# and Tn3#). CTX-M-15 was suggestively acquired with ISEcp1 element (100% identity with previously described ISEcp1 common region).

In addition, pKpnU95 carried multiple accessory regions that encode virulence and environmental persistence loci, including an extra citrate-dependent iron acquisition system *fecIRABCDE*, copper-silver resistance operons and a pair of UV resistance genes *umuCD* duplicating the one presented on the chromosome (Table 3). The increased tolerance of the clinical strain towards copper ions was demonstrated experimentally and supports the role of the plasmid-mediated copper resistance (Figure 2B).

To confirm the presence of pKpnU95 in the cured and reconstituted strains, we created a multi-PCR screen consisting of seven genes that are equally distributed throughout the plasmid. The screen includes an IncFIB(K) replicon and two main resistance genes CTX-M-15 and *qnrS1* together with persistence genes *chrA*, *pcoB*, *umuD* and *hisP* encoding histidine transport ATP-binding protein (Table S3 and Figure S1).

To study the self-transferability of pKpnU95 we performed conjugation experiments into two recipient *K. pneumoniae* strains: the cured strain, KpnΔpKpnU95, and *K. pneumoniae* rifampicin-resistant KPTA29 [39]. The conjugation was unsuccessful, supporting the absence of conjugation genes, except for a pseudogene *traI.* OriT was also not predicted from the plasmid sequence. In addition, the plasmid did not encode any complete toxin/antitoxin system possessing a single antitoxin gene *relB* and a putative, only computationally predicted, YafO family toxin (Table 3).

### 3.5. Analysis of pKpnU95 Closely Related Global Plasmids

To examine the uniqueness of pKpnU95, we retrieved all the complete sequence homologous plasmids from the Nucleotide collection (nt/nr) using BLASTn. The most closely related plasmid (pKpnU95 coverage, 68%) was plasmid unnamed2 carried by *K. pneumoniae* ST252 strain FDAARGOS_566 from a human origin (CP033758) [40]. To identify additional possible homologous plasmids in the Nucleotide database, we defined pKpnU95 backbone region (19,808 bp), which encodes IncFIB(K) replicon, plasmid stability proteins ParA and ParB and the lac operon. This backbone (coverage 100% and identity >99%) was found in two additional *K. pneumoniae* complete plasmid sequences, isolates FDAARGOS_439 plasmid unnamed1 (CP023917.1, pKpnU95 coverage 63%) and QMP_B2-170 plasmid unnamed (CP031799.1, pKpnU95 coverage 63%).

Given that pKpnU95 lacked Tra genes, we hypothesized that it is specifically associated with ST1412 genetic lineage. To support our hypothesis, we searched the INSDC for ‘*K. pneumoniae* ST1412’ but did not retrieve any available sequences. We then proceeded with our search in the NCBI sequence read archive (SRA) which retrieved five ST1412 isolates, which all belonged to a large *K. pneumoniae* clinical collection from Houston Methodist Hospital, isolated during 2011–2017 (*n* = 1964, BioProject accession numbers, PRJNA396774 and PRJNA376414, [41]). Four out of these five ST1412 isolates harbored sequences that showed high coverage to pKpnU95 (82–97% coverage breadth). To track the possible dissemination of pKpnU95 in the Houston isolate collection, we searched all WGS isolates (*n* = 1964) and found 11 additional *K. pneumoniae* isolates from other genetic lineages that encoded pKpnU95 backbone (100% coverage and >99% identity). Read mapping of these isolates with pKpnU95 sequence revealed 67 to 84% coverage.

**Figure 4:**
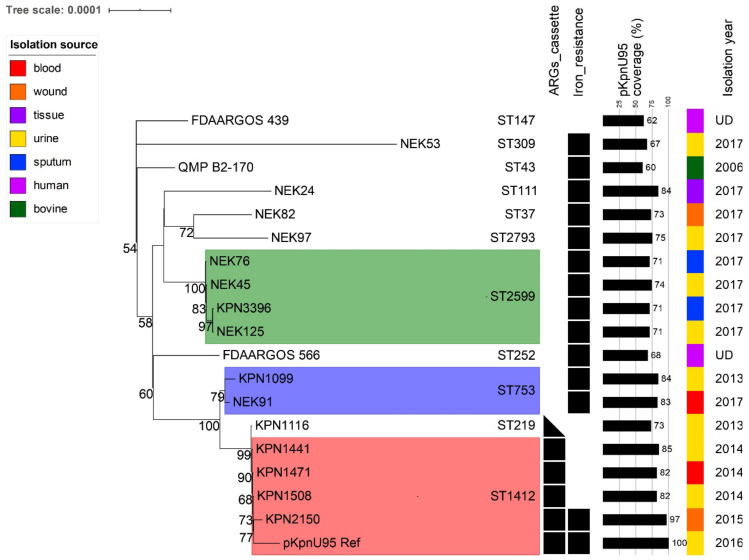
Phylogeny of pKpnU95-related plasmids. The maximum-likelihood tree shows the relationships between concatenated SNPs extracted from pKpnU95 and its related plasmids (*n* = 18). Bootstrap support values (>50% out of 1000 replicates) are presented at nodes. Plasmid groups carried by *K. pneumoniae* isolates of the same ST are highlighted with colors. Plasmid characteristics such as the presence of specific resistance cassettes and pKpnU95 coverage together with host STs, sources and years of isolations are presented on the right.

To study the clonal distribution of the 18 global pKpnU95-related plasmids (15 from Houston collection and three from the NCBI Nucleotide collection), we tracked their bacterial host sequence and capsule types. All the 18 plasmids were carried by *K. pneumoniae* that belonged to 11 different sequence types (ST1412-*n* = 4; ST2599-*n* = 4; ST753-*n* = 2; and single plasmids of distinct STs, Figure 3 and Figure 4). Four out of the five Houston *K. pneumoniae* ST1412 isolates that carried pKpnU95-related plasmid sequences possessed capsule type KL107. The remaining ST1412 isolate that lacked pKpnU95 (pKpnU95 coverage 1.7%) differed in its capsule type (KL7).

We generated a genetic map of pKpnU95 and the 18 related plasmids (Figure 3). The presence of accessory regions varied among the plasmids. The copper-silver resistance operons were identified in all the plasmids. The iron acquisition system (*fec* operon) was found in the majority of the plasmids (13/18) and other metal and UV radiation resistance genes appeared less: chromate (9/18), arsenic (7/18) and UV (6/18). Of all the related plasmids, *K. pneumoniae* ST1412 plasmids shared the highest genetic content, including the complete 10 ARGs resistome of pKpnU95 (Table 3) and a small multidrug resistance efflux transporter QacΔE1. The ARGs were surrounded by mosaic IS-containing regions, suggesting multiple genetic acquisition events. This resistome region was absent in non-ST1412 plasmids.

### 3.6. Co-Evolution of pKpnU95-Related Plasmids and Their K. pneumoniae Clonal Lineages Hosts

To study the evolutionary dynamics of plasmid pKpnU95, we assessed the core SNP-based plasmid phylogeny for pKpnU95 and the 18 *K. pneumoniae* global related plasmids that were included in the genomic comparison (Figure 4). Plasmid phylogeny revealed plasmid–bacterial host adaptation and demonstrated high conservation of plasmids carried in *K. pneumoniae* isolates of the same sequence type: ST1412, ST753, ST2599. The plasmid pKpnU95 showed the highest relatedness to four plasmids carried in *K. pneumoniae* ST1412, suggesting the co-evolution of IncFIK plasmids within distinct clonal lineages independent with their clinical origin.

## 4. Discussion

In this study, we performed a comprehensive genetic and physiological characterization of a clinical ESBL-producing *K. pneumoniae* ST1412 strain that caused a UTI in a healthy woman in the community setting.

As far as we know, KpnU95 is the first reported ST1412 community-associated isolate. Previous reports on this *K. pneumoniae* ST were all from hospital settings located in diverse geographical locations such as China [42], Tunisia [43] and in the United States [41].

As a primary pathogen causing a UTI in a healthy woman, we suspected KpnU95 to exhibit enhanced virulence. KpnU95 chromosomal virulome encompasses all the expected *K. pneumoniae* uropathogenic virulence factors, including type 1 and 3 adhesion pili, allantoin metabolism pathway, capsule and biofilm formation and the most common *K. pneumoniae* iron-acquisition system, enterobactin [4,6,44]. Interestingly, KpnU95 chromosome also encodes receptors for two hypervirulent siderophore systems, *iutA* (aerobactin) and *iroN* (salmochelin) [4,45]. It was suggested previously that these receptors enable a pathogen to intercept siderophores from co-existing bacteria [11]. KpnU95 virulome also encompassed a plasmid-encoded citric-dependent iron-acquisition system, *fecABCDIR* [46].

In order to evaluate the virulence of the CA-UTI KpnU95 isolate, we used the host–pathogen *C. elegans* model. KpnU95 caused complete nematode death in several hours. Our data form the first study that experimentally describes the virulence of *K. pneumoniae* ST1412 lineage. A comparison of its virulence phenotype with other reported *K. pneumoniae* virulence studies [47,48] is limited due to differences in the in vivo virulence assays used. Considering that KpnU95 was isolated from urine, we assessed the strain virulence in artificial urine and revealed that despite its tardy growth, the strain preserved its nematode killing ability, demonstrating its pathogenesis in urine conditions. This study is the first to describe the assessment of bacterial virulence using artificial urine in the *C. elegans* model and these conditions may be adopted further for UTI causing pathogens.

KpnU95 possessed a wide resistome, which consisted of both chromosomal and plasmid-mediated genes that supported its antibiotic resistance profile. The main contributor to the MDR phenotype of KpnU95 was its single megaplasmid, pKpnU95, which encoded ten ARGs, including *bla*_CTX-M-15_ and the plasmid-mediated quinolone resistance gene *qnrS1*. The combination of these genes has been described previously in other *K. pneumoniae* plasmids [49]. Interestingly, the chromosomal antimicrobial genetic determinants included the multidrug transporters co-encoded by *nfsA*, the clinically relevant nitroreductase gene that has been shown previously to confer resistance to nitrofurantoin [50]. The combination of ESBL, *qnrS1* and the intermediate resistance to nitrofurantoin pose a clinical challenge as CA-UTI are often treated empirically with either quinolones or nitrofurantoin as first-line treatment [51].

Antimicrobial resistance and virulence genes in *K. pneumoniae* are well documented as these are characteristic features for the identification of *Klebsiella* subpopulations such as hypervirulent and MDR strains [3,52]. However, physiologically essential genes whose role in *K. pneumoniae* population should be considered are persistence genes, which may contribute to the bacterial survival under environmental stresses, but unnecessarily impose a fitness cost on the carrying host. The combination of persistence genes may be termed ‘persistome’.

KpnU95 encompasses a wide array of persistome, including genes that encode for UV resistance and resistance to disinfectants and toxic metals such as copper, silver, arsenic and tellurite. Bacterial copper-resistance systems may be beneficial for uropathogenic *K. pneumoniae* strains as they may protect the bacterium from a higher urinary copper content [53]. KpnU95 also carried metal transporters allowing it to maintain a required concentration of magnesium [54], nickel, cobalt [55], zinc [56] and manganese [57].

In order to evaluate the role of pKpnU95 in the fitness and virulence of its carrying host, we cured the plasmid from the clinical KpnU95 strain and reconstituted the cured strain with pKpnU95 to study loss and gain of resistance and virulence rendered by the plasmid. The fitness cost of pKpnU95 was apparently undetectable in rich or minimal media, but in artificial urine, the plasmid provided some growth advantage. The absence of fitness cost may suggest plasmid–host adaption [58]. Moreover, the lack of conjugation genes in pKpnU95 supported its non-transmissible nature, further supporting its adaptation to ST1412 lineage. The presence or absence of pKpnU95 did not influence KpnU95 virulence in the *C. elegans* model, signifying the major role of the chromosomally encoded traits in the strain pathogenesis.

The complete sequence of pKpnU95 that we resolved allowed us to perform a global search for homologous plasmids, which were all found to be carried in *K. pneumoniae*, suggesting a narrow plasmid host range. Phylogenetic analysis of these plasmids revealed lineage-associated clustering. pKpnU95 showed the highest similarity with ST1412 plasmids alongside with higher pKpnU95 sequence coverage (82–97%). Nevertheless, a pKpnU95 ARG-encoding region (Figure 3) was found only in ST1412 lineage. Plasmids that are more distant were found in other *K. pneumoniae* STs with lower pKpnU95 sequence coverage (60–84%).

Mining available databases for pKpnU95 closely related plasmids was challenging. We first screened the complete sequence database, which did not reveal highly similar sequences, and then extended our search to the SRA database, which consists of raw WGS Illumina-based data. Due to the large size of the SRA and the difficulties in data processing, we limited our analysis only to ST1412-containing collections. Mining raw Illumina data expanded our search for homologous sequences and enabled us to track isolates that harbor pKpnU95-related plasmids in the Houston collection [41]. However, short read assembly limited our ability to restore the entire bacterial genomes and to prove similar plasmid structure [59].

pKpnU95 is an example of a MDR plasmid where resistance, virulence and persistence genes are co-selected. Co-selection of antibiotic and heavy metal resistance genes was previously described in another CTX-M-15-encoding IncF plasmid (pUUH239.2) isolated from an in-hospital outbreak-causing *K. pneumoniae*. This latter plasmid that encoded silver and arsenate resistance genes together with ARGs, when transformed into *E. coli* MG1655, was beneficial for the bacterial host in complex multidrug environments [60]. Another large plasmid co-encoding a wide range of ARGs (26 genes) and persistence genes (silver, copper, arsenic and mercury resistances and iron uptake) is sul1-sul2-sul3 and qnrB2-encoding IncF plasmid pR46-270 carried in *K. pneumoniae* ST37. This latter isolate was recovered from a rabbit that expands the reservoir of clinically essential plasmids to animal-derived bacteria [61].

In summary, this study presents the genomic analysis of an ESBL-producing MDR CA-UTI *K. pneumoniae* KpnU95 from an uncommon lineage ST1412. This strain carried a physiologically important MDR plasmid, pKpnU95. We characterized KpnU95 virulence features and confirmed them using the *C. elegans* host–pathogen model, and were able to demonstrate the role of the plasmid in bacterial fitness and persistence. Understanding *K. pneumoniae* resistance, virulence and persistence features will further assist in developing new therapeutic approaches.

## Figures and Tables

**Table 1:** Antibiotic susceptibilities of all the study strains.

Antibiotic Agent	MIC (μg/mL)				
	KpnU95	KpnΔpKpnU95 ^1^	KpnΔpKpnU95/pKpnU95 ^2^	*E. coli* DH10B	*E. coli* DH10B/pKpnU95 ^3^
Ampicillin	>16	>16	>16	<8	>16
Piperacillin	>64	<16	>64	<16	>64
Cefazolin	>16	2	>16	4	>16
Ceftazidime	16	<1	16	<1	16
Ceftriaxone	>32	<0.5	>32	<0.5	>32
Ampicillin/Sulbactam	16/8	8/4	16/8	<4/2	16/8
Piperacillin/Tazobactam	<16/4	<8/4	<8/4	<8/4	<8/4
Ticarcillin/ Clavulanic acid	32/2	<8/2	32/2	<8/2	32/2
Aztreonam	>16	<1	>16	<1	16
Imipenem	<0.5	<0.5	<0.5	<0.5	<0.5
Ertapenem	<0.25	<0.25	<0.25	<0.25	<0.25
Meropenem	<0.5	<0.5	<0.5	<0.5	<0.5
Doripenem	<0.5	<0.5	<0.5	<0.5	<0.5
Amikacin	<8	<8	<8	<8	<8
Tobramycin	<2	<2	<2	<2	<2
Gentamicin	<2	<2	<2	<2	<2
Ciprofloxacin ^4^	0.38	0.032	0.38	0.002	0.064
Nitrofurantoin	64	64	64	<32	<32
Trimethoprim/ Sulfamethoxazole	>4/76	<2/38	>4/76	<2/38	>4/76
Tetracycline	<4	<4	<4	<4	<4
Minocycline	2	2	2	<1	<1
Tigecycline	<1	<1	<1	<1	<1
Fosfomicin ^5^	≤16	≤16	≤16	≤16	≤16

^1^ The cured KpnU95 strain lacking pKpnU95. ^2^ The cured KpnU95 transformed with pKpnU95. ^3^ *E. coli* DH10B transformant carrying pKpnU95. ^4^ MIC determined by Etest. ^5^ Susceptibility determined using VITEK^®^2 system and interpreted according to CLSI guidelines [36].

**Table 2:** KpnU95 chromosomal virulence and antibiotic resistance genes.

Category	Functional Group	Genes ^1^	Function/Resistance to
Virulence	Fimbria adhesins	*fimBEAICDFGHK*	Type 1 fimbria
	Fimbria adhesins and biofilm formation	*mrkABCDFJIH*	Type 3 fimbria
		*ecpRABCDE*	*E. coli* common pilus
		*pilABC, pilQONM, pilT*	Type VI pilus
	Secretion systems (SS)	*gspCDEFGHIJLMN, pulS, pulO*	Type II SS
		*tssBCKDHIMAFGJ, tssE*	Type VI SS
	Toxins–antitoxin systems	*doc-phd, hipAB, ratAB, RelE-like TA*	Type II TA
		*YkfI-YfjZ*	Type IV TA
		*ecnAB*	Lipoprotein toxin entericidin
	Siderophores	*entABCDEFHS*^2^, *fepABCDGS*^2^	Enterobactin
	Siderophore receptors	*iutA, iroN*	Aerobactin and Salmochelin receptors
	Iron-acquisition and transport	*msrQ, hmuRS, foxA, cirA, fhuD, yfeX, feoABC, efeO*	heme-binding subunit, iron-acquisition proteins, hemin transport proteins
	Outer membrane porins	*ompA, ompC, ompF, ompN, ompW*	Iron uptake
	Transcriptional regulators	*fur*	Ferric iron uptake transcriptional regulator
		*rcsA, rscB*	Regulators of capsule synthesis/iron uptake
	Host-associated metabolism	*ureDABCEFG, urtABCDE*	Urease, urea ABC transporter
		*hpxB, hpxDE, hpxR, hpxO hyuE, pucU, codB*	Allantoin/purine metabolism and transport
	Virulence factors	*pgaABCD*	Biofilm synthesis
		*msgA*	Intracellular survival
		*hha*	Hemolysin expression modulator
		*mviMN, yihY*	Putative virulence factors
Persistence	Heavy metal resistance and homeostasis	*chrAB*	Chromate resistance and transport
		*cpxRA, cusF, cutE, cutC, cutF, cueO, cueR, copA, copCD, scsABCD*	Copper-binding response regulator systems, copper-binding protein, copper homeostasis, copper resistance
		*cusRS*	Copper/silver response regulator system
		*zraPSR*	Zinc/copper resistance
		*corA, corB, corC* *mgtA, mgtE*	Magnesium-cobalt transporters Mg/Co/Ni transporter
		*rcnA, rcnB*	Nickel/cobalt efflux Nickel/cobalt homeostasis
		*cbtJKL,* *cbiMNQO*	Cobalt ABC transporter, cobalt ECF transporter
		*nikABCDER, dppBC, oppDF*	Nickel ABC transporter
		*mntH, mntS, mntP, mntR* *sitABC, mtsC*	Manganese efflux pump, transport regulator, manganese ABC transporters
		*znuABC, zupT, zntB, zitB, fieF, zur, zntR*	Zinc transporters/uptake regulators
		*zntA,* *czcD*	Zn(II)/Cd(II)/Pb(II) translocating P-type ATPase, Cobalt-zinc-cadmium resistance protein/cation transporter
	Metalloid resistance	*arsRBC, yffB*	Arsenate reductase, regulator, arsenic efflux pump
		*tehAB, ydcL, yebE*	Tellurite resistance
		tsgA	Putative selenite and tellurite transporter
	UV	*umuCD*	Error-prone, lesion bypass DNA polymerase V
Antibiotic resistance	ARG	*blaSHV-1*	Penicillin
		*fosA*	Fosfomycin
		*nfsA*	Nitrofurantoin
	Multidrug transporters	*oqxAB*	Quinolone resistance
		*macAB*	Macrolide resistance
		*mexB*	Acriflavine resistance
		*eefABC*	Tolerance response to inorganic acid
		*acrAB, acrD, acrEF, tolC*	Resistance to beta-lactams, aminoglycosides, fluoroquinolones, tetracycline, chloramphenicol, acriflavine
		*mdtABCD, mdtG, mdtH, mdtJI, mdtK, mdtL, mdtM, mdtNOP, mdtQ*	Transporters of wide range of multidrug and disinfectant components ^3^
		*emrRAB, emrD*	Resistance to nalidixic acid, thiolactomycin, novobiocin Participates in a low energy shock adaptive response
	Transcriptional activators	*marRAB, soxRS, ramA, rarA*	Activation of both antibiotic resistance and oxidative stress genes

^1^ Present in the order of CDSs. ^2^ Random order. The actual order of the CDS was *entHABEC, fepB, entS, fepDGC, entF, fepSA, entD*. ^3^ Including acriflavine, chloramphenicol, norfloxacin, enoxacin, novobiocin, fosfomycin and deoxycholate, SDS, ethidium bromide and tetraphenylphosphonium bromide.

**Table 3:** Accessory genes encoded on pKpnU95.

Category	Functional Group	Genes ^1^	Function/Resistance to
Antibiotic resistance	ARG	*bla*_CTX-M-15_	ESBL
		*mph(A)*	Macrolides
		*strA, strB, aadA2, aph(3′)-Ia*	Aminoglycosides
		*sul1, sul2*	Sulphonamides
		*qnrS1*	Quinolones
		*dfrA12*	Trimethoprim-sulfa
	Multidrug transporter	*qacEΔ1 (emrE)*	Small multidrug resistance (SMR) efflux transporter
Virulence	Iron	*fecIRABCDE*	Ferric citrate transport system
Persistence	Heavy metal resistance and homeostasis	*chrA*	Chromate transport
		*pcoABCDRS, copA, copG*	Copper resistance Metal-binding protein
		*cusCFBA*	Copper-silver efflux system proteins
		*cusRS,* *pcoE (silE),* *silP*	Copper-silver response regulator system, copper-silver binding protein, Ag(+)/copper-translocating P-type ATPase
	Metalloid resistance	*arsRBC, arsH*	Arsenate reductase, arsenic resistance, regulator, arsenic efflux pump
	Toxins-antitoxin	*relB, yafO-like* ^2^	Antitoxin and putative toxin
	Others	*cld*	Chlorite dismutase
	UV	*umuCD*	Error-prone, lesion bypass DNA polymerase V

^1^ Present in the order of CDSs. ^2^ Computationally predicted only.

## Data Availability

KpnU95 WGS data and pKpnU95 are available at GenBank (BioProject PRJNA494961) and at the NCBI Nucleotide database (GenBank accession number MK552109), respectively.

## Supplementary Materials

The following are available online at https://www.mdpi.com/article/10.3390/microorganisms9051022/s1. Table S1: Average generation times of KpnU95, the cured, and the plasmid reconstituted cured strain, Table S2: Average generation times of KpnU95, the cured, and the plasmid reconstituted cured strain in the presence of copper, Table S3: PCR primers to screen the presence of pKpnU95 accessory unique genes encoded on pU95, Figure S1: PCR screening for the presence of pKpnU95 plasmid in the different studied strains, Figure S2. ERIC-PCR analysis of KpnU95 strains.
