# Supplementary material for: Genomics and Virulence of Klebsiella pneumoniae Kpnu95 ST1412 Harboring a Novel Incf Plasmid Encoding Blactx-M-15 and Qnrs1 Causing Community Urinary Tract Infection
Source: Microorganisms. 2021 May 10;9(5):1022. doi: 10.3390/microorganisms9051022 (PMC8151138; doi:10.3390/microorganisms9051022)
Supplement: Supplementary file 1 [file microorganisms-09-01022-s001.zip › microorganisms-1194752-supplementary.pdf]

microorganisms

Supplementary

Genomics and Virulence of *Klebsiella Pneumoniae* Kpnu95 ST1412 Harboring a Novel Incf Plasmid Encoding *Bla*CTX-M-15 and *Qnrs1* Causing Community Urinary Tract Infection

Ayala Gancz [1], Kira Kondratyeva [1], Dorit Cohen-Eli [1] and Shiri Navon-Venezia [1,2,*]

[1] Molecular Biology Department, Faculty of Life Sciences, Ariel University, Ariel 40700, Israel; ayalagancz@gmail.com (A.G.); lutra007@gmail.com (K.K.); dorit553@gmail.com (D.C.-E.)
[2] The Miriam and Sheldon Adelson School of Medicine, Ariel University, Ariel 40700, Israel
* Correspondence: shirinv@ariel.ac.il

Citation: Gancz, A.; Kondratyeva, K.; Cohen-Eli, D.; Navon-Venezia, S. Genomics and Virulence of *Klebsiella Pneumoniae* Kpnu95 ST1412 Harboring a Novel Incf Plasmid Encoding *Bla*CTX-M-15 and *Qnrs1* Causing Community Urinary Tract Infection. *Microorganisms* **2021**, *9*, 1022. https://doi.org/10.3390/microorganisms9051022

Academic Editor: Jane Turton

Received: 7 April 2021
Accepted: 4 May 2021
Published: 10 May 2021

Publisher's Note: MDPI stays neutral with regard to jurisdictional claims in published maps and institutional affiliations.

Copyright: © 2021 by the authors. Licensee MDPI, Basel, Switzerland. This article is an open access article distributed under the terms and conditions of the Creative Commons Attribution (CC BY) license (http://creativecommons.org/licenses/by/4.0/).

1. Supplementary Materials

Table S1. Average generation times of KpnU95, the cured, and the plasmid reconstituted cured strain

K. pneumoniae isolate	Average generation time ± SD (min) in different media		
	LB	BM2	Artificial urine
KpnU95 (Clinical)	42.21 ± 0.50	73.45 ± 2.01	95.37 ± 2.09
KpnU95ΔpKpnU95 (Cured)	41.82 ± 0.88	73.64 ± 2.22	103.85 ± 2.68*
KpnU95ΔpKpnU95/pKpnU95 (Cured reconstituted)	40.94 ± 1.69	73.44 ± 1.06	89.99 ± 7.50

* Significantly long generation time of KpnU95ΔpKpnU95 versus KpnU95 clinical strain (*p* value = 0.0361) using Student's t-test.

Table S2. Average generation times of KpnU95, the cured, and the plasmid reconstituted cured strain in the presence of copper

K. pneumoniae isolate	Average generation time ± SD (minutes) in different $CuSO_4$ concentration [mM], *p* value			
	0	2	4	8
KpnU95 (Clinical)	51.93 ± 0.005	50.53 ± 0.48	54.56 ± 1.35	78.85 ± 3.87
KpnU95ΔpKpnU95 (Cured)	52.14 ± 4.12	57.41 ± 0.35**	63.33 ± 1.18*	86.29 ± 2.77
KpnU95ΔpKpnU95/pKpnU95 (Cured reconstituted)	51.93 ± 0.03	52.46 ± 0.18	62.59 ± 0.002*	76.25 ± 0.34

* $p \leq 0.05$ and ** $p \leq 0.005$ versus KpnU95 with Student's t-test.

Table S3. PCR primers to screen the presence of pKpnU95 accessory unique genes encoded on pU95.

Primer	Sequence (5′ – 3′)	Target	Amplicon Size (bp)	Reference
CTX-M1-F<br>CTX-M1-R	AAAAATCACTGCGCCAGTTC<br>AGCTTATTCATCGCCACGTT	*bla*$_{CTX-M-group1}$	415	[1]
umuD_ pU95_F<br>umuD_ pU95_R	ATGTTCTTAATTCCAATGGAAAATCC<br>TTACAGATTACCCCGGGCA	*umuD*	423	This study
IncF(K)_pU95_F<br>IncFIB(K)+partRep_pU95_R	GATCATTCGCTCGATGTCTG<br>CCCGCTGATGAGTTTGGGAT	*IncFIB(K)*	571	This study
qnrS1_ pU95_F<br>qnrS1_ pU95_R	ATGGAAACCTACAATCATACATAT CG<br>TTAGTCAGGATAAACAACAATACCCA	*qnrS1*	657	This study
HisP_pU95_F<br>HisP_pU95_R	ATGCGTGATTATGCTATTGAG<br>TCATGCTGACTCCTTCAATGC	*hisP*	819	This study
chrA_pU95_F<br>chrA_pU95_R	ATGAACGATACTGCCAGGA<br>TCACAGTGCTAAACTCAACAAC	*chrA*	1206	This study
pcoB_pU95_F<br>pcoB_pU95_R	ATGCTGTTGAAAACGTCTCG<br>TCATTCCTCCACCCGGACTT	*pcoB*	1818	This study

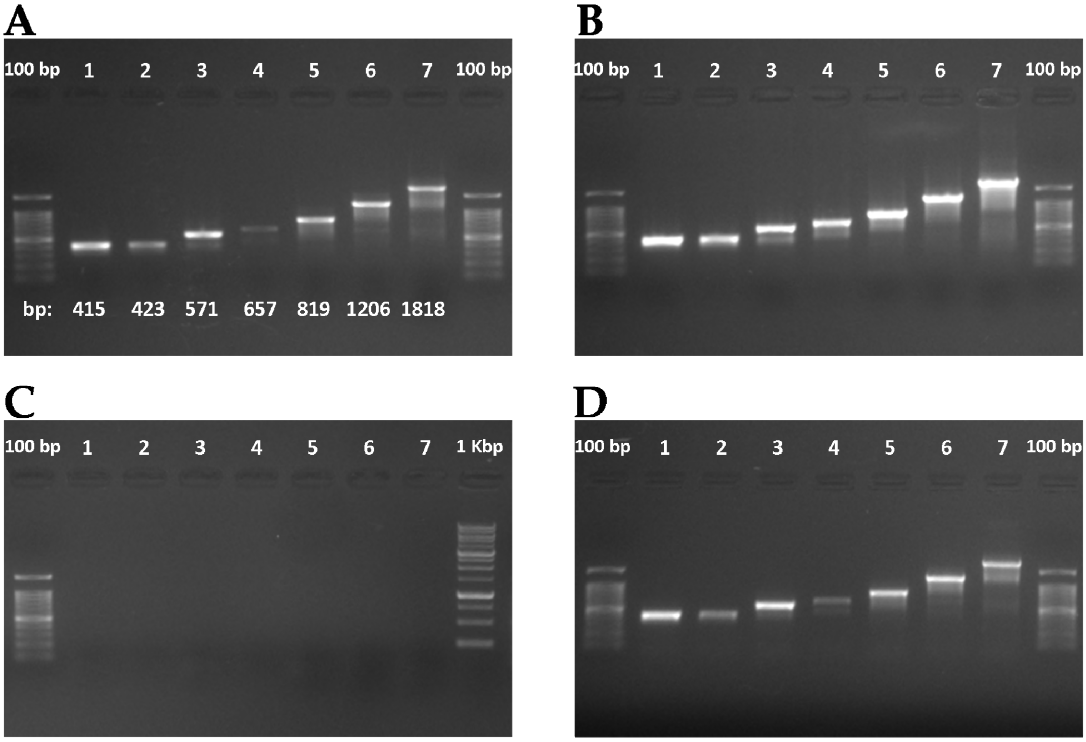

Figure S1. Targeted PCR screening for validating the presence/absence of pKpnU95 plasmid in the different studied strains. The PCR amplified seven genes encoded on pKpnU95. The expected amplicons are presented in the clinical KpnU95 strain (amplicon sizes appear in base pairs, bp) (**A**); PCR performed on the purified plasmid pKpnU95 (**B**); lysate of the cured strain KpnΔpKpnU95 (**C**), and from the reconstituted strain KpnU95-cured/pKpnU95 (**D**). The seven pKpnU95 amplified genes in panels **A-D** are: lane 1 - *bla*$_{\text{CTX-M-group1}}$; lane 2- *umuD*; lane 3 - IncFIB(K); lane 4 - *qnrS1*; lane 5 - *hisP*; lane 6 - *chrA*; lane 7 - *pcoB*. The DNA molecular weight markers are 1kb and 100bp ladders (GeneDirex). Electrophoresis was performed in 1% agarose.

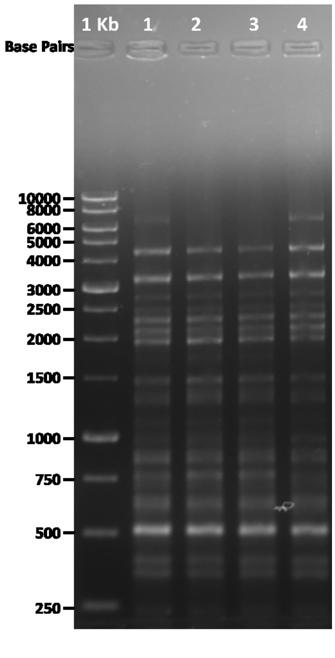

Figure 2. ERIC-PCR analysis of KpnU95 strains. ERIC-PCR pattern of crude DNA lysates of KpnU95, lane 1; KpnU95 after passages at 42^{0}C as a control, lane 2; KpnΔpKpnU95, lane 3, and KpnΔpKpnU95/pKpnU95, lane 4. The DNA molecular weight marker is a 1kb ladder (Bio-Labs). Electrophoresis was performed in 1% agarose gel.

References

1. Woodford, N.; Fagan, E.J.; Ellington, M.J. Multiplex PCR for rapid detection of genes encoding CTX-M extended-spectrum β-lactamases. *J. Antimicrob. Chemother.* **2006**, *57*, 154–155.
